# *Leishmania mexicana* Induces Limited Recruitment and Activation of Monocytes and Monocyte-Derived Dendritic Cells Early during Infection

**DOI:** 10.1371/journal.pntd.0001858

**Published:** 2012-10-18

**Authors:** Patricia M. Petritus, Daniel Manzoni-de-Almeida, Ciara Gimblet, Claudia Gonzalez Lombana, Phillip Scott

**Affiliations:** 1 Department of Pathobiology, School of Veterinary Medicine, University of Pennsylvania, Philadelphia, Pennsylvania, United States of America; 2 Nucleo de Pesquisa em Ciências Biológicas, Universidade Federal de Ouro Preto, Ouro Preto, Brazil; University of Edinburgh, United Kingdom

## Abstract

While C57BL/6 mice infected in the ear with *L. major* mount a vigorous Th1 response and resolve their lesions, the Th1 response in C57BL/6 mice infected with *L. mexicana* is more limited, resulting in chronic, non-healing lesions. The aim of this study was to determine if the limited immune response following infection with *L. mexicana* is related to a deficiency in the ability of monocyte-derived dendritic cells (mo-DCs) to prime a sufficient Th1 response. To address this issue we compared the early immune response following *L. mexicana* infection with that seen in *L. major* infected mice. Our data show that fewer monocytes are recruited to the lesions of *L. mexicana* infected mice as compared to mice infected with *L. major*. Moreover, monocytes that differentiate into mo-DCs in *L. mexicana* lesions produced less iNOS and migrated less efficiently to the draining lymph node as compared to those from *L. major* infected mice. Treatment of *L. mexicana* infected mice with α-IL-10R antibody resulted in increased recruitment of monocytes to the lesion along with greater production of IFN-γ and iNOS. Additionally, injection of DCs into the ear at the time of infection with *L. mexicana* also led to a more robust Th1 response. Taken together, these data suggest that during *L. mexicana* infection reduced recruitment, activation and subsequent migration of monocytes and mo-DCs to the draining lymph nodes may result in the insufficient priming of a Th1 response.

## Author Summary

Leishmaniasis, caused by protozoan parasites belonging to the genus, *Leishmania*, exhibits clinical symptoms ranging from mild cutaneous lesions to more severe cutaneous or visceral disease. Here, we focus on *L. major* and *L. mexicana*, two species that lead to self-resolving and chronic cutaneous lesions, respectively. A strong Th1 response is necessary for resolution of disease following *L. major* infection. However, *L. mexicana* infection induces a limited Th1 response resulting in chronic disease. Monocyte-derived dendritic cells are believed to be important in priming the Th1 response during L. major infection, and therefore in this study we evaluated whether there are quantitative and/or qualitative differences in monocyte-derived dendritic cells following *L. mexicana* infection. We found that fewer monocytes were recruited to the lesions of *L. mexicana* infected mice as compared to mice infected with *L. major*. In addition, there were fewer iNOS producing monocyte-derived dendritic cells in the lesions of *L. mexicana* infected mice and less migration of monocyte-derived dendritic cells to the draining lymph node. Manipulations that allow for increased monocytes in the lesions of *L. mexicana* infected mice also resulted in a more robust Th1 response. Thus, these findings provide a mechanistic basis for the limited Th1 response observed during *L. mexicana* infection and also offer a better understanding of the important role that monocytes play during infection with *Leishmania*.

## Introduction

Infection with *Leishmania* results in a variety of outcomes, depending on the parasite species and immune response mounted by the host [1]. Murine disease models resemble human disease, with some infections being self-healing and others chronic. Resolution of leishmaniasis requires the production of IFN-γ by Th1 cells; the absence of a strong Th1 response results in chronic disease with non-healing lesions [2], [3]. Th1-mediated protection is promoted by IFN-γ-induced production of nitric oxide (NO) in infected cells, which ultimately leads to parasite killing [2], [3]. In C57BL/6 mice, infection with *L. major* results in a strong Th1 response with self-resolving lesions, in contrast, *L. mexicana* lesions fail to resolve [4]. The chronic nature of *L. mexicana* lesions is most likely due to their inability to stimulate an effective Th1 response [4], [5], [6]. Similarly, *L. amazonensis* fails to induce a strong Th1 response and leads to chronic lesions in mice [7], [8], [9], [10]. However, the immune mechanisms limiting Th1 responses following either *L. mexicana* or *L. amazonensis* infection are not yet fully defined.

Several demonstrations that infection with *L. mexicana* suppresses IL-12 production by macrophages and dendritic cells (DCs) [11], [12], [13] suggested that failure to produce IL-12 may limit the Th1 response, resulting in the observed susceptibility to *L. mexicana* [14], [15], [16]. However, we found that administration of IL-12 failed to promote disease resolution, suggesting that the inability of *L. mexicana* mice to resolve their infection is not solely dependent upon lack of IL-12 [17]. Therefore, we hypothesized that a more generalized deficit in DC function may contribute to the chronic lesions that develop following *L. mexicana* infection.

Monocyte-derived DCs (mo-DCs) play an important role in the development of protective immunity [18], [19], [20], [21]. Mo-DCs differentiate from inflammatory monocytes (CD11b^+^, Ly6C^+^, CCR2^+^ and CX3CR1^lo^) recruited to sites of inflammation. Once activated, mo-DCs produce inducible nitric oxide synthase (iNOS) [22]. Indeed, mo-DCs appear to be the major producers of iNOS during *L. major* infection [23] and are therefore likely essential for reducing the parasite burden.

In addition to iNOS production, mo-DCs contribute to immunity following infection with *L. major* by migrating to draining lymph nodes (dLN) where they stimulate antigen-specific Th1 T cell responses [24]. Moreover, we recently found that *L. major*-activated DCs induce lymph node hypertrophy, which promotes additional recruitment of naïve T cells into the lymph node to enhance the protective response [25]. Taken together, these data indicate that mo-DCs play an important role in the development of protective immunity to. *L. major*, and that a deficit in their recruitment or activation might limit a protective Th1 response.

In the present study, we investigated whether the meager Th1 response observed during *L. mexicana* infection is due to limitations in the: 1) recruitment of monocytes from the blood to the site of infection; 2) differentiation of monocytes into iNOS-producing mo-DCs; and/or 3) migration to the draining lymph node. We found that monocyte recruitment to the site of infection was reduced in *L. mexicana* infected mice compared to *L. major* infected mice. Moreover, while monocytes in *L. mexicana* lesions upregulated expression of CD11c, they produced significantly less iNOS and migrated less efficiently to the draining lymph node relative to monocytes in *L. major* infected mice. Following treatment of *L. mexicana* infected mice with α-IL-10R antibody, there was increased recruitment of monocytes to the lesion, as well as increased production of IFN-γ and iNOS. Additionally, when DCs were injected into the ear at the time of infection with *L. mexicana*, there was a more robust Th1 response. These data imply that the poor Th1 response observed during *L. mexicana* infection results from both reduced monocyte recruitment to the lesions, and a relative deficit in their differentiation into functional effector populations.

## Materials and Methods

### Ethics Statement

All animal studies were carried out in compliance with the guidelines of the Institutional Animal Care and Use Committee (IACUC) of the University of Pennsylvania and in accordance with the recommendations in the Guide for the Care and Use of Laboratory Animals of the National Institutes of Health. The animal protocol was approved by the IACUC of the University of Pennsylvania, Philadelphia PA.

### Mice, Parasites and Infections

Female C57BL/6 (B6) and B6-Ly5.2/Cr (CD45.1) mice were purchased from the National Cancer Institute (Fredricksburg, MD). Animals were maintained and experiments were carried out in a specific pathogen-free environment. *L. major* V1 parasites (MHOM/IL/80/Friedlin) or *L. mexicana* parasites (MNYC/BZ/62/M379) were grown until stationary phase in Schneider's Drosophila medium (Gibco, Grand Island, NY) supplemented with 20% heat-inactivated FBS (Gibco) and 2 mM l-glutamine (Sigma). Metacyclic promastigotes were isolated by density gradient [26]. For infection of mice, 1×10^5^ metacyclic parasites were injected into the ear.

### Flow cytometry

For flow cytometry, cells were isolated from ears or draining lymph nodes. Dermal ear sheets were separated and incubated in Liberase TL (Roche, Indianapolis, IN) for 1 hr at 37°C. Ears and draining lymph nodes were made into single cell suspensions and washed with PBS. Fixable Aqua dye (Invitrogen, Carlsbad, CA) was added to assess cell viability. Cells were then incubated with Block (CD16/32, inactivated mouse sera and Rat IgG) followed by fluorochrome-conjugated antibodies for surface markers (CD11c, CD11b, CD45.1, CD45.2, MHCII (e-Bioscience, San Diego, CA), and Ly6C (BD Pharmingen, San Diego, CA). Intracellular staining was performed for iNOS using an unconjugated anti-iNOS/NOS II rabbit polyclonal IgG (Millipore, Temecula, CA) followed by flourochrome-conjugated donkey-anti-rabbit IgG (e-Bioscience). Briefly, surface-stained cells were fixed in PBS with 2% paraformaldehyde and then permeabilized with 0.2% saponin in FACS staining buffer (PBS containing 0.1% BSA). Cells were fixed by using 2% paraformaldehyde and samples were acquired on a FACS Canto flow cytometer (BD Pharmingen). Analysis was performed using FlowJo software (Tree Star, Ashland, OR).

### Monocyte purification and transfer

Bone marrow was harvested from tibias and femurs of naïve mice. Following lysis of red blood cells with ACK lysis buffer (Lonza, Walkersville, MD), Miltenyi MACS columns were used to purify monocytes. Briefly, anti-Ly6G-biotin antibody and biotin microbeads (Miltenyi, Auburn, MD) were added to the cells, which were then placed over a LS column. Ly6G^+^ cells on the column were discarded and anti-PE CD11b (e-Bioscience) and PE microbeads were added to the flow through, which was placed over a 2^nd^ LS column. CD11b^+^ cells attached to the column were washed off. Monocytes were enriched to 50% as evaluated by flow cytometry. 1×10^6^ total cells from the final column were transferred into mice by intradermal injection into the lesions.

### FITC painting

Mice were anesthetized using ketamine/xylazine and the ventral side of each ear was painted with FITC isomer (Sigma, St. Louis, MO). FITC (8 mg/mL) was dissolved in an equal volume of acetone and dibutyl phthalate (Sigma) and 25 µL of the mixture was applied to the skin. Migration of FITC^+^ cells was assessed in the draining lymph node 48 hours following application.

### Anti-IL-10R Treatment

Mice were injected intraperitoneally with 500 µg of α-IL-10R antibody (1B1.3A, BioXcell, West Lebanon, NH) one day prior to intradermal infections of 1×10^5^ *L. mexicana* metacyclics in both ears. Mice were subsequently treated with 500 µg of α-IL-10R antibody on day 3 and then 250 µg every 3 days until the final harvest at 2 weeks post-infection.

### ELISA

Supernatants from draining lymph node cultures stimulated with *L. mexicana* freeze-thaw antigen for 72 hrs were collected and assayed by sandwich ELISA using paired monoclonal antibody to detect IFN-γ.

### Generation of dendritic cells and transfer

DCs were generated as previously described [27]. Briefly, bone marrow cells from C57BL/6 mice were isolated from femurs and tibias of mice by syringe flushing. Bone marrow cells were counted and seeded into 6-well plates at 5×10^5^ cells/mL in 3 mLs of media - RPMI 1640 (Gibco) supplemented with 10% heat-inactivated FBS (Gibco), 2 mM glutamine (Sigma), 50 µM 2-ME (Gibco), 100 U/mL penicillin (Sigma), 100 µg/mL streptomycin (Sigma) and 20 ng/mL GM-CSF (Peprotech, Rocky Hill, NJ) per well. Cells were maintained at 37°C with 5% CO_2_ and fed on days 3, 6 and 8 with 3 mLs of fresh media. Cells were harvested on day 10 and injected into the ear of C57BL/6 mice at the time of infection. Briefly, 1×10^6^ DCs and 1×10^5^ *L. mexicana* metacyclics were mixed immediately prior to injecting into the ear.

### Statistics

Statistical significance was determined using unpaired, two-tailed Student's *t* test. Results with a p value ≤0.05 were considered significant.

## Results

### Fewer monocytes are recruited during infection with *Leishmania mexicana* compared to *L. major*

**Figure 1:**
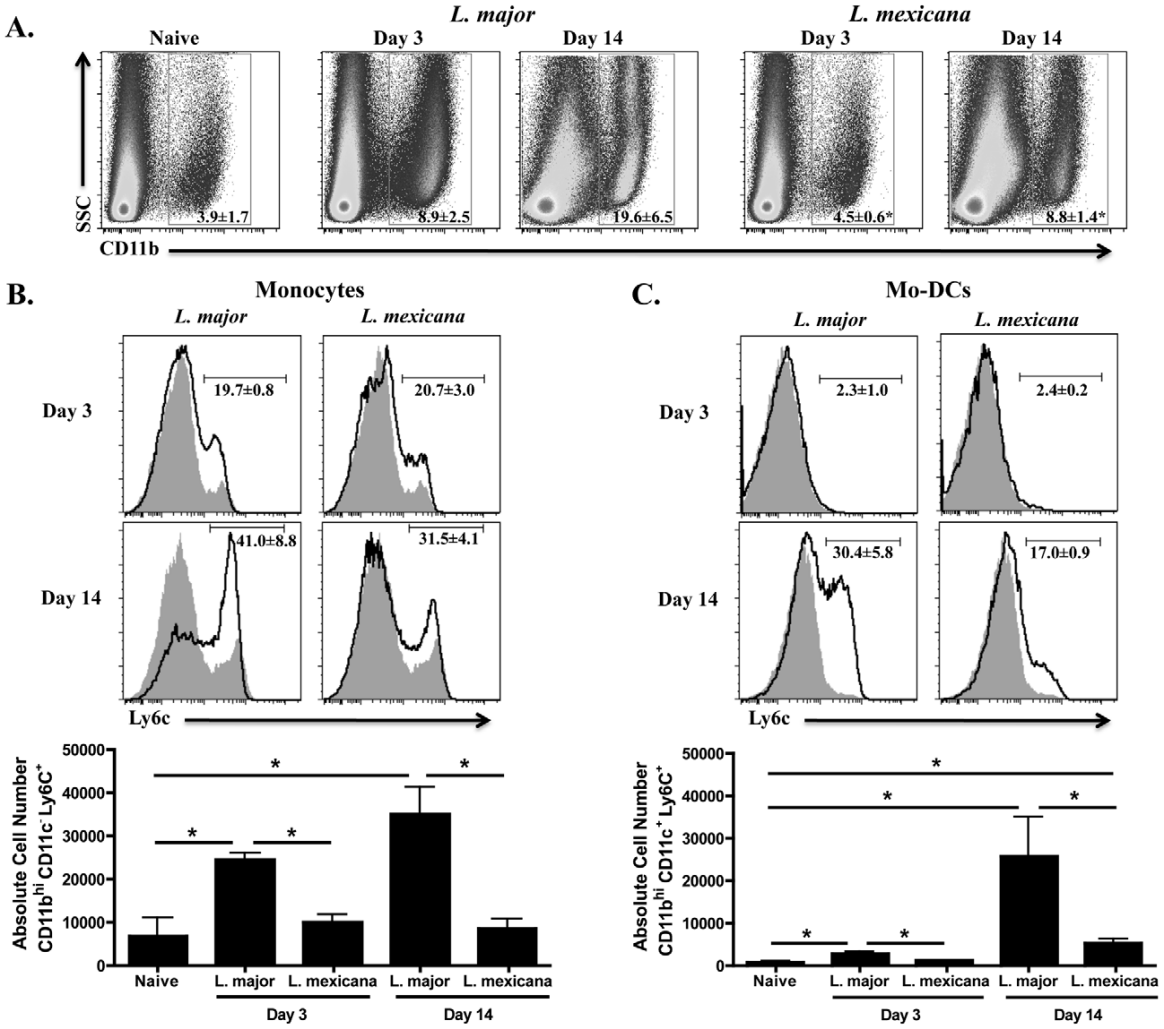
Fewer CD11b^hi^ Ly6C^+^ cells in the ear following *L. mexicana* infection compared to *L. major*. Ears from naïve, as well as *L. major* or *L. mexicana* infected C57BL/6 mice were processed to single cell suspensions. (**A**) Percentage of CD11b^hi^ cells present in the ear of naïve or infected mice on day 3 and 14. Cells are previously gated on total, single live cells. Histograms of monocytes (**B**) or mo-DCs (**C**) in the ear of naïve (grey shaded histogram) or infected mice (black line) on day 3 and 14 and absolute number of monocytes (**B**) or mo-DCs (**C**) from naïve, day 3 and day 14 infected mice. Monocytes are pre-gated on CD11b^hi^ CD11c^−^ cells and mo-DCs are previously gated on CD11b^hi^ CD11c^+^ cells. The results expressed are the mean percentage (± SD for FACS plots) or the mean number of cells (± SE for bar graphs) of 3 mice per group. The results are representative of two experiments. * significantly lower (p<0.05) compared to *L. major* infected mice in Fig. 1A or p<0.05 between indicated groups in Fig. 1B and C.

The development of a protective response following *L. major* infection is associated with the recruitment of monocytes into the lesion, which are believed to differentiate into mo-DCs (defined as CD11b^hi^, CD11c^+^, Ly6C^+^) to prime a strong Th1 response [24]. Since *L. mexicana* infection promotes chronic, non-healing lesions and a minimal Th1 response, we hypothesized that fewer monocytes would be recruited to lesions following infection with *L. mexicana* compared to *L. major*. To test this, we infected C57BL/6 mice with either *L. major* or *L. mexicana* parasites and assessed the cellular composition of the lesions at 3 and 14 days post-infection. Expression of CD11b, a subunit of α_M_β_2_ (also known as Mac-1 and CR3), was used to detect infiltrating leukocyte populations, including monocytes, macrophages, and granulocytes [28]. At 3 days post-infection, there was a significant increase in the percentage of CD11b^hi^ cells in dermal lesions from *L. major* infected mice compared to normal skin (Fig. 1A). In contrast, no increase in CD11b^hi^ cells was observed in lesions from *L. mexicana* infected mice. Moreover, the difference in percentage of CD11b^hi^ cells between *L. major* and *L. mexicana* lesions was still evident two weeks after infection (Fig. 1A). In contrast, neutrophil (CD11b^hi^ Ly6G^+^) frequency increased equally in lesions of both *L. major* and *L. mexicana* infected mice by day 14 as compared to normal skin (data not shown). Consistent with the observed alterations in CD11b^hi^ cells, there was an increase in inflammatory monocytes (CD11b^hi^ CD11c^−^ Ly6C^+^) in the lesions from 3-day and 14-day *L. major* infected mice as compared with normal skin (Fig. 1B) while no such increase was observed following *L. mexicana* infection (Fig. 1B). By day 14, mo-DCs (CD11b^hi^ CD11c^+^ Ly6C^+^) were evident in lesions of both *L. major* and *L. mexicana* infected mice, however, mo-DCs were preferentially represented in *L. major* lesions (Fig. 1C). Taken together, these results suggest that *L. mexicana* fails to promote the recruitment of monocytes, reducing the number of cells available for subsequent differentiation into mo-DCs capable of controlling parasite numbers.

### *L. mexicana* lesions have fewer iNOS producing mo-DCs compared to *L. major*

**Figure 2:**
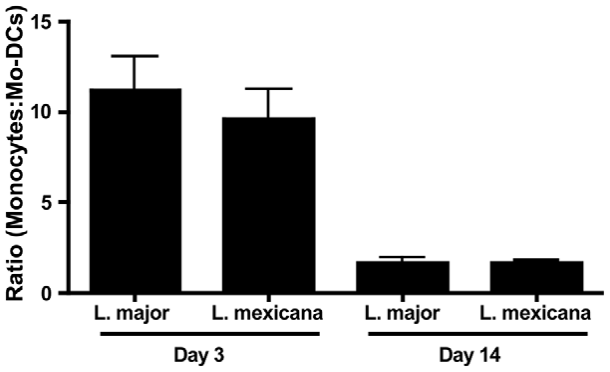
*L. mexicana* infection does not lead to a defect in differentiation of monocytes to mo-DCs. C57BL/6 mice were infected in the ear with *L. major* or *L. mexicana* and ears were processed as above. Percentage of monocytes and mo-DCs on day 3 and 14 were used to determine a ratio of differentiation from monocytes to mo-DCs in *L. major* or *L. mexicana* infected mice. The results expressed are the mean ratio (± SE) of 3 mice per group. The results are representative of two experiments.

**Figure 3:**
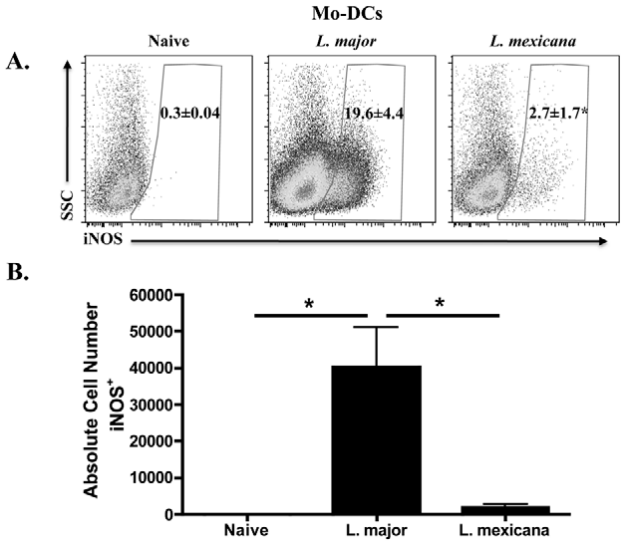
Mo-DCs produce significantly less iNOS during infection with *L. mexicana* compared to *L. major*. C57BL/6 mice were infected and ears were processed as above. In addition to staining for surface markers, intracellular staining for iNOS was performed. Percentage (**A**) or absolute number (**B**) of iNOS-producing cells in the ear of naïve or infected mice on day 14. Cells are previously gated on live, singlets that are CD11b^hi^ CD11c^+^. The results expressed are the mean percentage (± SD for FACS plots) or the mean number of cells (± SE for the bar graph) of 3 mice per group. The results are representative of two experiments. * significantly lower (p<0.05) compared to *L. major* infected mice.

Although there were fewer monocytes recruited to lesions from *L. mexicana* infected mice compared with those from *L. major* infected mice, the ratio of monocytes to mo-DCs was similar (Fig. 2). In these experiments we did not determine if the CD11c^+^ Ly6C^+^ cells were derived from monocytes, but based on previous findings [24], this is our assumption. However, mo-DCs in the lesions from *L. mexicana* infected mice expressed significantly less iNOS compared with mo-DCs from *L. major* lesions. Thus, while approximately 20% of the mo-DCs in lesions from *L. major* infected mice were iNOS^+^, only 3% were iNOS^+^ in lesions from *L. mexicana* infected mice (Fig. 3A). Similarly, the number of iNOS-producing mo-DCs was significantly reduced in lesions from mice infected with *L. mexicana* (Fig. 3B). CD11b^hi^ CD11c^−^ Ly6C^+^, inflammatory monocytes, did not make iNOS in either *L. major* or *L. mexicana* infected mice (data not shown). These data demonstrate that there are fewer iNOS-producing mo-DCs in *L. mexicana* infected mice, potentially contributing to the inability of these mice to resolve their infection.

### Migration of mo-DCs from lesions to the draining lymph node occurs less efficiently during *L. mexicana* infection compared to *L. major*

In addition to killing parasites at the site of infection through iNOS-dependent mechanisms, mo-DCs also migrate to dLNs where they orchestrate the developing immune response through antigen presentation and regulation of cytokine production [29]. Recently, we have also shown that *L. major*-activated DCs promote lymph node hypertrophy following infection [25] and the impaired lymph node expansion following *L. mexicana* infection [30] led us to investigate if a reduction in mo-DCs migration to the draining lymph node during *L. mexicana* infection limits the Th1 response and impairs lymph node expansion.

**Figure 4:**
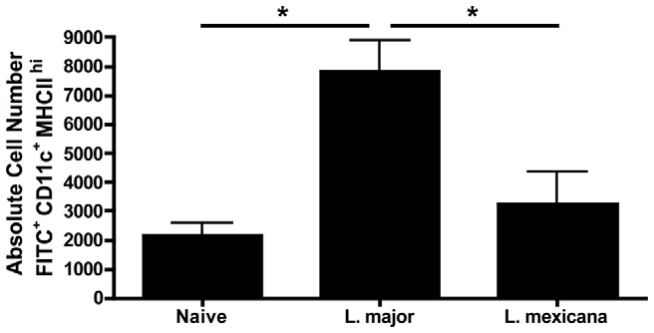
Fewer FITC+ endogenous mo-DCs migrate to the dLN during *L. mexicana* infection compared to *L. major*. C57BL/6 mice were infected as above for two weeks. FITC isomer was applied to the ears of naïve, *L. major* or *L. mexicana* infected mice on day 14 and dLNs were harvested and processed forty-eight hours later. FITC^+^ MHCII^hi^ CD11c^+^ cells were enumerated in the dLNs. These cells are previously gated on live, singlets that are CD11b^hi^. The results expressed are the mean number of cells (± SE) of 3 mice per group. The results are representative of two experiments. * significantly different (p<0.05) between indicated groups.

To evaluate the ability of DCs to migrate from the site of infection, C57BL/6 mice were infected in the ear with *L. major* or *L. mexicana* and two weeks post-infection the ears were FITC painted. After 48 hours we compared the FITC^+^ DCs (CD11c^+^ MHCII^hi^) in the draining lymph node from naïve, *L. major* infected or *L. mexicana* infected mice. Notably, there were significantly more FITC^+^ DCs in *L. major* infected mice when compared to either naïve or *L. mexicana* infected mice. In contrast, there was no difference in the number of FITC^+^ DCs between naïve and *L. mexicana* infected mice (Fig. 4), indicating that mo-DCs migration to the dLN is compromised in *L. mexicana* infected mice.

**Figure 5:**
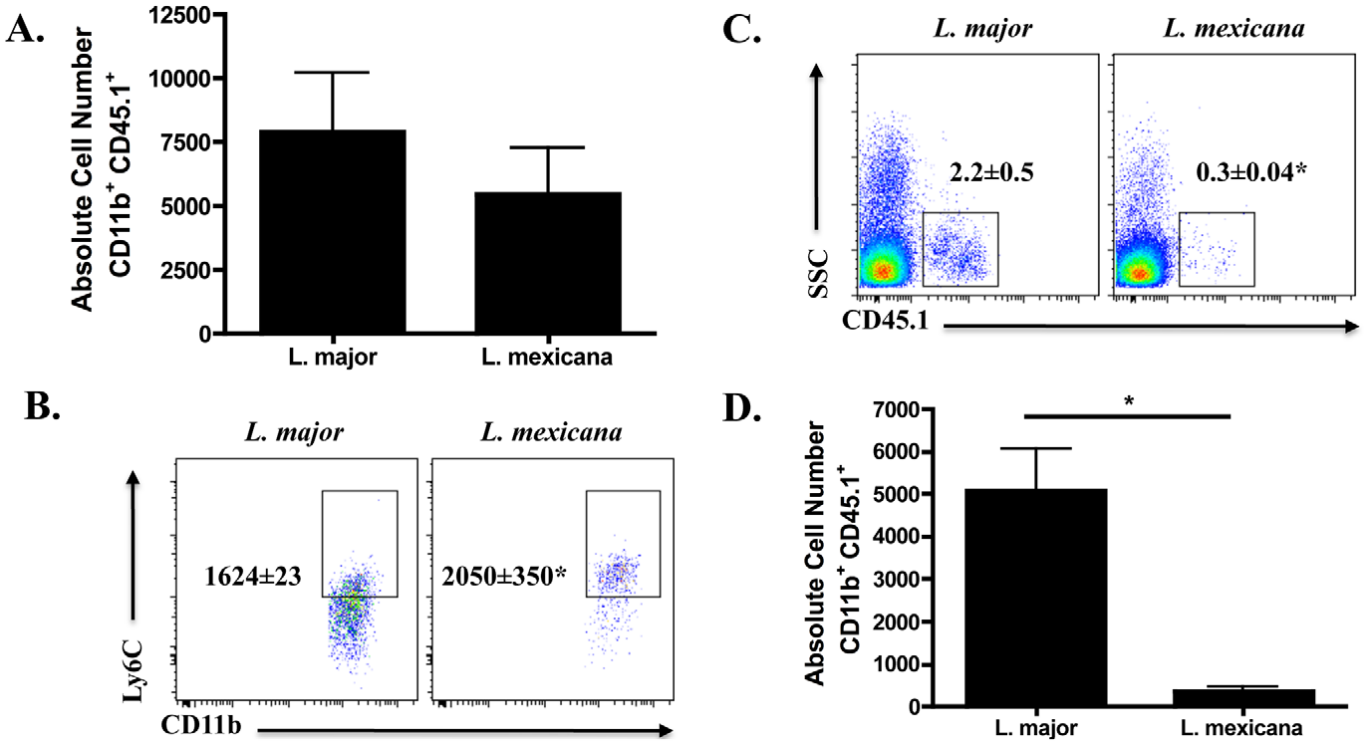
Fewer transferred monocytes migrate to the dLN during *L. mexicana* infection compared to *L. major*. Monocytes enriched from CD45.1 C57BL/6 mice were injected into the ear of CD45.2 C57BL/6 mice that were infected for two weeks with either *L. major* or *L. mexicana*. Eighteen hours following injection of the monocytes, ears and dLNs were harvested and processed. (**A**) Absolute number of transferred monocytes (CD11b^+^ CD45.1^+^) in the ear. Cells are previously gated on total, live cells that are singlets. (**B**) Mean fluorescence intensity (MFI) of Ly6C on transferred cells recovered from the ears of infected mice. Percentage (**C**) or absolute number (**D**) of CD45.1^+^ cells in the dLN. These cells are previously gated on live, singlets that are CD11b^hi^ cells. The results expressed are the mean percentage (± SD for FACS plots) or the mean number of cells (± SE for bar graphs) of 3 mice per group. The results are representative of two experiments. * significantly different (p<0.05) compared to *L. major* infected mice.

We next wanted to determine if the microenvironment within *L. mexicana* lesions actively inhibited DC migration. Therefore, we injected the same number of CD45 disparate monocytes into *L. major* or *L. mexicana* lesions and evaluated their migration to the draining lymph node. Figure 5A shows an equivalent number of CD11b^+^ CD45.1^+^ cells in the ear of *L. major* or *L. mexicana* infected mice approximately 18 hours following monocyte transfer. Interestingly, the expression of Ly6C on the donor monocytes was lower in *L. major* infected mice as compared to *L. mexicana* infected mice (Fig. 5B). As downregulation of Ly6C is associated with activation of mo-DCs [24], [31], these data suggest that mo-DCs in *L. mexicana* infected mice do not differentiate as efficiently as mo-DCs from *L. major* infected mice. Even more strikingly, there is a dramatic increase in both the frequency and absolute number of transferred cells in the draining lymph node of *L. major* infected as compared to *L. mexicana* infected mice (Fig. 5C and D), indicating that *L. mexicana* infection does not increase mo-DC trafficking to dLNs. However, we cannot exclude the possibility that there may be a difference in retention in the dLN of *L. major* versus *L. mexicana* infected mice. Together, these data indicate that a lack of mo-DCs migration from the site of *L. mexicana* infection to the draining lymph node may prevent T cell priming and impair lymph node expansion, precluding the induction of a protective Th1 response and resulting in the development of chronic disease.

### IL-10 limits monocyte recruitment during infection with *L. mexicana*

**Figure 6:**
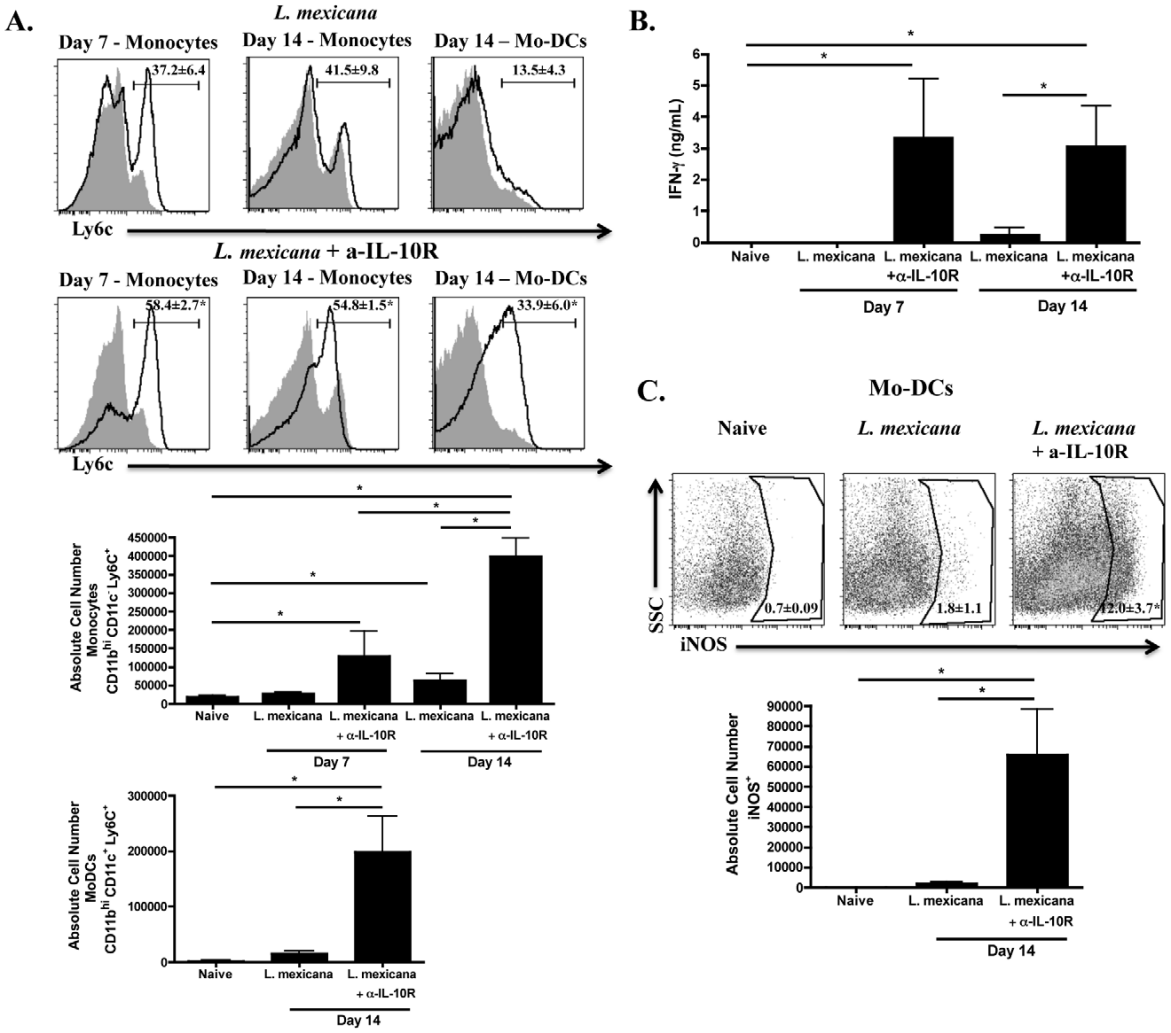
Production of IL-10 during *L. mexicana* infection contributes to less recruitment of monocytes. Ears from naïve, *L. mexicana* infected or *L. mexicana* infected and α-IL-10R treated C57BL/6 mice were processed as above. (**A**) Histograms of monocytes on day 7 and 14 or mo-DCs on day 14 in the ear of naïve (grey shaded histogram), *L. mexicana* infected mice (black line, top) or *L. mexicana* infected and α-IL-10R treated mice (black line, bottom). Absolute numbers of monocytes and mo-DCs are also shown. Monocytes are previously gated on live, singlets that are CD11b^hi^ CD11c^−^. Mo-DCs are previously gated on live, singlets that are CD11b^hi^ CD11c^+^. (**B**) Levels of IFN-γ (ng/mL) from the supernatants of single cell suspensions from the dLN of each group that were stimulated for 72 hours with *L. mexicana* freeze-thaw antigen. (**C**) Percentage and absolute number of iNOS-producing cells in the ear of naïve, *L. mexicana* infected mice or *L. mexicana* infected and α-IL-10R treated mice on day 14. Cells are previously gated on live, singlets that are CD11b^hi^ CD11c^+^. The results expressed are the mean percentage (± SD for FACS plots) or the mean number of cells (± SE for bar graphs) of 3–5 mice per group. The results are representative of two experiments. * significantly higher (p<0.05) compared to *L. mexicana* infected mice in Fig. 6A and 6C (FACS plots) or p<0.05 between indicated groups in Fig. 6A, 6B and 6C (bar graphs).

IL-10 has been described as having anti-inflammatory effects during infection by inhibiting cytokine production and antigen presentation [32], however, more recently it was shown that IL-10 also limits recruitment of CD11b^+^ Ly6C^+^ monocytes following *T. brucei* infection [33]. Since IL-10^−/−^ mice infected with *L. mexicana* resolve their lesions [6], we wanted to investigate whether blocking interaction of IL-10 with its receptor would lead to increased monocyte recruitment. We infected C57BL/6 mice as before with *L. mexicana* and treated one group with α-IL-10R antibody. We evaluated monocyte recruitment to lesions on days 7 and 14 following infection and found that there was a greater percentage and number of monocytes recruited to *L. mexicana* lesions in mice treated with α-IL-10R (Fig. 6A). Similarly, the percentage and number of mo-DCs in the lesions of *L. mexicana* infected mice was also significantly increased when IL-10R was blocked (Fig. 6A). Moreover, there were increased levels of IFN-γ in the draining lymph nodes (Fig. 6B), as well as a greater percentage and number of iNOS-producing mo-DCs in the lesions of *L. mexicana* infected mice treated with α-IL-10R (Fig. 6C). These data suggest that IL-10 is a key factor contributing to the limited number of monocytes observed during *L. mexicana* infection since blocking the interaction of IL-10 with its receptor results in a dramatic increase in monocytes and mo-DCs in the lesion. Surprisingly, we did not see a difference in the parasite burden in treated and untreated *L. mexicana* infected mice at this early time point, in spite of the fact that we have previously shown that IL-10^−/−^ mice eventually resolve their *L. mexicana* lesions [6]. Our assumption is that the effect on parasite burden is simply delayed and will develop later.

### Injecting DCs into the ear at the time of infection with *L. mexicana* results in a more robust Th1 response

**Figure 7:**
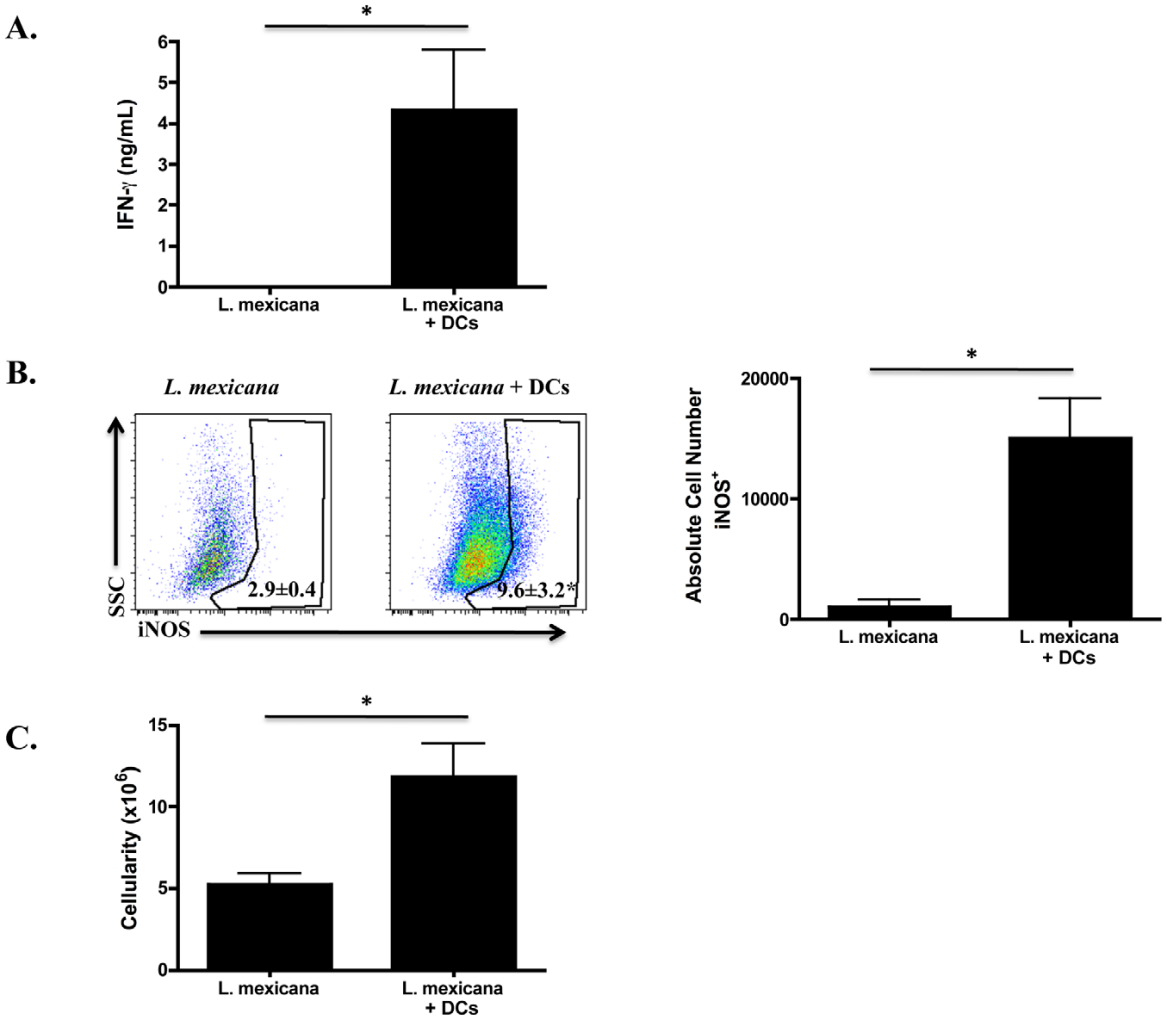
DCs injected at the time of infection with *L. mexicana* lead to a more robust Th1 response. C57BL/6 mice were infected as before in the ear with *L. mexicana*, however, one group also received DCs that were derived in vitro. At two weeks post-infection ears and dLNs were processed. (**A**) Levels of IFN-γ (ng/mL) from the supernatants of single cell suspensions from the dLN of each group that were stimulated for 72 hours with *L. mexicana* freeze-thaw antigen. (**B**) Percentage and absolute number of iNOS-producing cells in the ear of *L. mexicana* infected mice or *L. mexicana* infected mice that received DCs. Cells are previously gated on live, singlets that are CD11b^hi^ CD11c^+^. (**C**) The cellularity of the dLN in *L. mexicana* infected mice or *L. mexicana* infected mice that received DCs. The results expressed are the mean percentage (± SD for FACS plots) or the mean number of cells (± SE for bar graphs) of 2–4 mice per group. The results are representative of two experiments. * significantly higher (p<0.05) compared to *L. mexicana* infected mice.

The previous experiment, where *L. mexicana* infected mice were treated with α-IL-10R antibody, suggests that the increase in mo-DCs in the lesion may result in the priming of an improved Th1 response. Here, we test whether there is a correlation between increased numbers of DCs in the lesion and a more robust Th1 response. We injected DCs into the ear at the time of infection with *L. mexicana* and we compared the Th1 response 14 days post-infection to *L. mexicana* infected mice receiving no DCs. As predicted, *L. mexicana* infected mice receiving DCs produced greater levels of IFN-γ (Fig. 7A), and had a greater percentage and number of iNOS-producing DCs (Fig. 7B). Moreover, the impaired lymph node expansion that occurs during infection with *L. mexicana* was overcome in mice that received DCs (Fig. 7C). Taken together, these data suggest that the limited Th1 response observed in *L. mexicana* infected mice can be overcome if a greater number of DCs can be established in the lesion.

## Discussion

Infection of C57BL/6 mice with either *L. major* or *L. mexicana* results in cutaneous lesions. However, while *L. major*-induced lesions heal, those induced by *L. mexicana* infection do not. The chronicity of *L. mexicana* infections is attributable to the limited Th1 response mounted by the host to the parasite [5], [6], [34]. Since the development of a Th1 response in leishmaniasis depends upon IL-12 production by DCs [35], [36], [37], [38], it was originally thought that *L. mexicana* fails to induce a healing response due to its inability to stimulate IL-12 production [11], [12], [14], [16]. However, the limited Th1 response in *L. mexicana* infected mice is not reversed by treatment with rIL-12 [17], suggesting that there is a more generalized impairment in DC function.

Differentiation of mo-DCs from inflammatory monocytes at the site of infection plays an essential role in immune protection in a number of infectious diseases [19], [21]. Monocytes are recruited to *L. major* infected skin [23], [24], [39] and mo-DCs are thought to be essential for the induction of the Th1 response in *L. major* infection [24], suggesting that limitations in monocyte recruitment and differentiation (or both) may lead to chronic disease following *L. mexicana* infection. In support, our current studies demonstrate that fewer monocytes are recruited during infection with *L. mexicana* when compared to *L. major*, and the consequent reduction in differentiated mo-DCs present in *L. mexicana* lesions likely compromises generation of a protective Th1 response. In addition, reduced monocyte recruitment and the observed decrease in iNOS expression will limit the killing capacity of these cells [39], presumably leading over time to increased parasite burden. Together, the limited Th1 response and enhanced parasite burden could promote the chronic exacerbated disease observed following *L. mexicana* infection.

The importance of monocyte recruitment in limiting the progression of infectious diseases has been most clearly demonstrated in CCR2 deficient (CCR2^−/−^) mice. CCR2 is a chemokine receptor expressed on inflammatory monocytes that mediates monocyte chemotaxis. In CCR2^−/−^ mice, Ly6C^hi^ monocytes accumulate in the bone marrow due to their inability to emigrate from this site. Limited recruitment of monocytes to the site of infection likely contributes to the enhanced susceptibility of CCR2^−/−^ mice to *Listeria* infection [40]. In addition, following oral *Toxoplasma gondii* infection of CCR2^−/−^ mice, monocytes fail to be recruited to the illeum, allowing for uncontrolled parasite growth. However, adoptive transfer of CCR2-expressing monocytes into *T. gondii* infected CCR2^−/−^ mice protected them from this otherwise lethal infection [41]. CCR2^−/−^ mice infected with *L. major* are also more susceptible to infection due to an attenuated Th1 response [42]. Interestingly, treatment of CCR2^−/−^ mice with rIL-12 is able to reverse the susceptibility to *L. major* infection [43]. Since mo-DCs have been described as the major producers of IL-12 during *L. major* infection [24], these data support our hypothesis that compromised recruitment of monocytes to the lesion influences the development of a Th1 response in *L. mexicana* infected mice.

As IL-10 has been shown to limit the recruitment of CD11b^+^ Ly6C^+^ monocytes during infection with *T. brucei* [33] and we have previously shown that IL-10^−/−^ mice infected with *L. mexicana* resolve their lesions [6], we hypothesized that monocyte recruitment following *L. mexicana* infection is impacted by IL-10 production at the lesion site. In fact, we showed that by blocking IL-10R, there was increased recruitment of CD11b^hi^ Ly6C^+^ monocytes to *L. mexicana* infected lesions. Moreover, *L. mexicana* infected mice treated with α-IL-10R produced significantly more iNOS and IFN-γ than *L. mexicana* infected C57BL/6 mice. As during *T. brucei* infection [33], it is likely that production of IL-10 in *L. mexicana*-induced lesions may work on several levels. IL-10 could lead to decreased levels of CCL2, which would explain the limited recruitment of monocytes into the lesions. IL-10 is also capable of dampening Th1 responses, which would result in lower levels of iNOS and IFN-γ. Therefore, these data provide a mechanism as to why there is limited recruitment of monocytes to the lesion during infection with *L. mexicana*.

Finally, while DCs are clearly needed to prime T cells in the draining lymph node, they also promote lymph node hypertrophy. We previously demonstrated that lymph node hypertrophy is associated with the protective response to *L. major* infection [30] and have more recently revealed that *L. major*-activated DCs are responsible for lymph node expansion [25]. During infection with *L. mexicana*, lymph node hypertrophy is greatly reduced, potentially limiting the immune response [30]. Here we have used two methods to track migration of mo-DCs from the lesion to the draining lymph node; one method marked endogenous mo-DCs in the lesion and the other utilized injection of CD45 disparate monocytes directly into the lesion. While fewer endogenous mo-DCs from the *L. mexicana* lesion migrated to the dLN as compared to *L. major*, this may have been due to the relatively low numbers of monocytes initially present within the lesions of *L. mexicana* infected mice. To address this problem, we injected equal numbers of monocytes into *L. major* or *L. mexicana* lesions, and found that there was still a deficit in the migration of mo-DCs to the dLN from *L. mexicana* lesions. An inability of mo-DCs to migrate to the dLN could prevent both antigen-specific responses, as well as mo-DC-driven lymph node hypertrophy, providing a potential explanation for the reduction in lymph node size in *L. mexicana* infected mice. Interestingly, if DCs are injected into the ear at the same time of infection with *L. mexicana*, mice have significantly larger lymph nodes and are able to mount a more robust Th1 response compared to mice that did not receive DCs. These data clearly demonstrate that mo-DCs are important in initiating an appropriate immune response against *Leishmania* and that the limited recruitment of monocytes observed during *L. mexicana* infection could lead to the chronic nature of the disease.

In summary, we have demonstrated that 1) fewer monocytes are recruited to lesion during infection with *L. mexicana* as compared to *L. major*, 2) fewer iNOS producing mo-DCs are present in the lesions of *L. mexicana* infected mice 3) fewer mo-DCs migrate to the dLN node during *L. mexicana* infection, 4) blocking IL-10R leads to increased monocyte recruitment and a more robust Th1 response during *L. mexicana* infection, and 5) injection of DCs into the ear at the time of infection with *L. mexicana* also leads to increased levels of iNOS and IFN-γ. Together, these findings provide a mechanistic basis for the limited Th1 response, and lack of lymph node hypertrophy observed in *L. mexicana* infected mice and offer a better understanding of the important role that monocytes play during infection with *Leishmania*.
